# HDFCN: A Robust Hybrid Deep Network Based on Feature Concatenation for Cervical Cancer Diagnosis on WSI Pap Smear Slides

**DOI:** 10.1155/2023/4214817

**Published:** 2023-04-17

**Authors:** Nitin Kumar Chauhan, Krishna Singh, Amit Kumar, Swapnil Baburav Kolambakar

**Affiliations:** ^1^USIC&T, Guru Gobind Singh Indraprastha University, New Delhi 110078, India; ^2^Department of ECE, Indore Institute of Science & Technology, Indore 453331, India; ^3^DSEU Okhla Campus-I, Formerly G. B. Pant Engineering College, New Delhi 110020, India; ^4^Department of Electronics Engineering, Indian Institute of Technology (BHU), Varanasi 221005, India; ^5^Bluecrest university college, Monrovia, Liberia

## Abstract

Cervical cancer is a critical imperilment to a female's health due to its malignancy and fatality rate. The disease can be thoroughly cured by locating and treating the infected tissues in the preliminary phase. The traditional practice for screening cervical cancer is the examination of cervix tissues using the Papanicolaou (Pap) test. Manual inspection of pap smears involves false-negative outcomes due to human error even in the presence of the infected sample. Automated computer vision diagnosis revamps this obstacle and plays a substantial role in screening abnormal tissues affected due to cervical cancer. Here, in this paper, we propose a hybrid deep feature concatenated network (HDFCN) following two-step data augmentation to detect cervical cancer for binary and multiclass classification on the Pap smear images. This network carries out the classification of malignant samples for whole slide images (WSI) of the openly accessible SIPaKMeD database by utilizing the concatenation of features extracted from the fine-tuning of the deep learning (DL) models, namely, VGG-16, ResNet-152, and DenseNet-169, pretrained on the ImageNet dataset. The performance outcomes of the proposed model are compared with the individual performances of the aforementioned DL networks using transfer learning (TL). Our proposed model achieved an accuracy of 97.45% and 99.29% for 5-class and 2-class classifications, respectively. Additionally, the experiment is performed to classify liquid-based cytology (LBC) WSI data containing pap smear images.

## 1. Introduction

Cancer is coerced by the obsolete and irregular evolution of cells in the human body. This deformity can infiltrate the nearby cells in that tissue together with the other tissues and may disperse into more body organs. Cervical cancer arises due to the contagion of the human papillomavirus (HPV) which causes an anomaly in the cervix through which the lower portion of the uterus and vagina connect. Cervical cancer was the fourth most genre of cancer in statics of new indices and fatalities following breast, colorectum, and lung cancer in 2020 [1]. The scarcity of screening and therapeutic systems consequences a high mortality rate in low and middle-income economies. The preliminary traits of cervical cancer comprise an erratic feminine cycle, postintercourse vagary bleeding, strong vaginal stink with discharge, inexplicable and relentless pelvic, intestinal, or back agony, exhaustion, and diminution in weight [2, 3].

An adequate diagnosis can be procured by using the potential preliminary investigation of cervical lesions for the contraction of the mortality rate by cause of cervical cancer. The most trusted and well-known approach for the detection of precancerous cells and cervical lesions is a Pap smear test [4]. The conventional and prevailing method for scrutinizing anomalies of peculiar cells of pap slides using a microscope by clinical experts is a quite sophisticated, tedious, and slow proceeding that requires decent knowledge and familiarization.

Artificial intelligence (AI) in computer vision is in growing vogue amid the researchers to go beyond the bounds of manual screening of pap smear data. Even though conventional machine learning (ML) models have reduced computing intricacy, they still require incorporating the extraction of features manually [5]. DL comes out as one of the fine automated end-to-end solutions to the multitudinous challenges in biomedical image processing [6–10]. To produce a decision support system with high efficacy and potential, a deep model requires plenty of medical image data for training. TL addresses this issue, by pretraining the model over a large amount of data followed by utilizing it with a limited image dataset of the specific problem [11].

**Figure 1:**
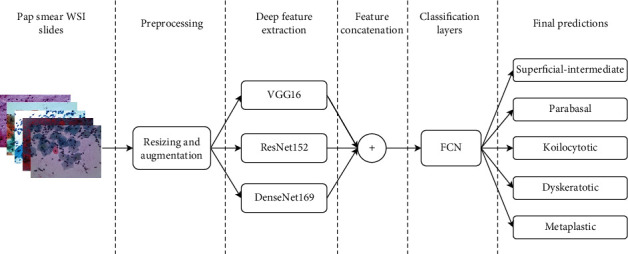
Structural outline of the proposed framework.

This paper proposes a novel approach that utilizes concatenated features for the classification of pap smear WSI images. Here, we used TL for the automatic retrieval of key features using fine-tuned deep models. Three fine-tuned DL models VGG-16, ResNet-152, and DenseNet-169 are used for feature extraction. These models use pretrained weights on the ImageNet dataset. The features retrieved from these individual models are concatenated and used for the prediction of test samples. A fully connected network (FCN) is used for classification by training it on concatenated features. The structural outline of the proposed framework is shown in Figure 1.

Commonly, the cells are first segmented from the WSI slides, and then the segmented cell images are used for the prediction of cancer. However, cell segmentation is a complex process in itself, and no algorithm performs equally well for cell segmentation from test slides obtained in distinct medical procedures and test conditions. Here, in this paper, we propose a model to analyze the multicell cervix images to detect the various grades of abnormality. The detection of abnormal samples from this proceeding enables other downstream investigations by segmentation of cells from abnormal WSI images.

The contribution of this research work is listed below:

1. A brief insight into current research in cervical cancer diagnosis, the use of intensive two-step augmentation, and the proposed HDFCN model to detect WSI pap-smear cervical images
2. The proposed method uses optimally extracted deep features to get concatenated into hybrid features that are used with FCN for test image classification
3. This proposed model gets validated on two WSI cervical cancer data—(1) SIPaKMeD data for 5-class and 2-class classifications and (2) LBC data classification
4. McNemar's statistical tool indicating *p* value is used to illustrate the demarcation of efficacy among the proposed classifier and the individual deep classifier utilized in the proposed hybrid model.

The remainder of the paper is articulated as follows: Section 2 contains the recent work related to cervical cancer diagnosis using different DL models.  Section 3 describes a detailed description of the proposed method and material utilized.  Section 4 depicts the result of the proposed model and discussion on various aspects of its performance. Lastly, Section 5 has some brief conclusions and the futuristic possibilities of the work.

## 2. Related Work

With the advancement of computer-aided diagnostic (CAD) tools, the use of DL has been motivated in the classification of cervix malignancies. Papers [12–16] give a detailed survey of the latest DL applications in cervigram histopathological image classification. The most renowned publicly available databases of cervical cancer are the Herlev [17], ISBI [18], Risk factor-based dataset [19], Cervix93 [20], SIPaKMeD [21], LBC dataset [22], etc.

In [23], 502 pathological images were collected at Xinjiang Medical University which were used to generate two groups of image datasets. The first group of 3012 images was generated by resizing and cropping, and the other group contains 108,432 images obtained using augmentation functions like rotation, flipping, and image enhancement on the original set of image data. A convolutional neural network (CNN) was used to perform 3-class classification with 93.33% and 89.48% accuracy for the first and second groups of images, respectively. In another work [24], a shallow CNN classifier was used for the classification of 684 positive and negative area handcrafted patches of 15 × 15 pixels retrieved from cervigram slides obtained by VIA of 102 women patients.

In this study [25], binary classification was performed using a stacked autoencoder followed by a softmax layer on the UCI dataset with 668 samples. The stack autoencoder and softmax layer were used for dimension reduction of raw data and classification, respectively, with 97.8% accuracy. In [26], TL used fine-tuning of the Inception-V3 model for the classification of 307 histopathology images collected by AQP, HIF, and VEGF staining at Shengjing Hospital of China Medical University. Original data got enhanced by 256 multiples, resulting in a total of 78,592 images using augmentation operations—rotation and flipping, provided a mean accuracy of 77.3% with the classifier model.

The authors [27] used four CNN classifiers, namely, AlexNet, DenseNet-121, ResNet-50, and GoogLeNet, to classify the Herlev dataset by combining the morphology and appearance-based features to provide topmost accuracy of 94.5%, 71.3%, and 64.5% for 2 class, 4 class, and 7 class, respectively, with the GoogLeNet classifier. In research [28], an approach using Mask R-CNN was used for the segmentation and classification of the Herlev database, using ResNet-10 as a key pillar. During segmentation, the proposed network was pretrained on the COCO dataset and provided a precision of 0.92 ± 0.06, recall of 0.91 ± 0.05, and ZSI of 0.91 ± 0.04. The VGG-like model was used for the classification of segmented cells with an accuracy of 98.1% for 2-class and 95.9% for 7 class. The work [29] introduced an AI classifier by modification of layers in the ResNet-50 model which classifies the colposcopy database of 310 images having 213 HSIL and 97 LSIL cases with an accuracy of 82.3%.

The experiment [30] proposed a pipeline of CNN-based feature extraction and classification networks founded on the identification of the cervix region of interest (ROI) and trained on two datasets: Intel & MobileODT Dataset and NCI Guanacaste Project Dataset. The developed model has a lightweight and faster framework and is found to be quite useful in mobile application development for low-income nations for the diagnosis of cervigram images. The work [31] presented ensemble learning and a CNN model and achieved an accuracy of 90.4% and 91.6%, respectively, for the two classifiers on the Herlev dataset with preprocessing methods. In [32], hybrid TL was implemented with pretrained AlexNet and VGG-16 models on 1644 cervix cell images collected at the National Institute of Health (NIH) and National Cancer Institute (NCI). This analysis provided a result of 91.46% accuracy for 2-class classification.

In the study [33], a deep network ColpoNet was introduced to detect colposcopy images collected at the National Cancer Institute (NCI) and achieved an accuracy of 81.35%, providing better efficiency than GoogleNet, AlexNet, ResNet-50, VGG-16, and LeNet. In the research [34], a deep framework containing seven networks, namely, ResNet-50, 5 incidents of ResNet-101, and a graph convolution network with edge traits (E-GCN), was introduced. The initial six networks were used for feature interpretation, and GCN classified the cervix images based on fused node and edge features. The proposed model evaluated a database with 7,688 colposcopic images and obtained an accuracy of 78.33% for positive and negative instance classification.

The work [35] implemented two classifiers TL-based VGG-19 and an ensemble model on colposcopy (CYENET) which were used to classify the colposcopy image data with 5,679 images collected by Intel & MobileODT and available at Kaggle. The proposed CYENET classifier gave a result of 92.3% with a 19% improvement in classification accuracy to VGG-19 (TL). In another study [36], the ResNet-50 model-based deep CNN (DCNN) model is used to perform classification besides three ML classifiers XGB, SVM, and RF, on cervicography data comprising 4,119 images with positive and negative incidences. Linear regression was applied to select 10 attributes out of 300; those were fed to the ResNet-50 classifier which performed better than others with accuracy and AUC of about 90.65% and 0.97, respectively.

In this experiment [37], CytoBrain was introduced which comprises a compact VGG network to identify the cervical cell from WSI slides. This model showed faster and more precision with an accuracy of about 88.3% on huge data containing 198,952 cervix cell images. In [38], a deep residual network was developed that assesses the performance with distinct activation functions like ReLU, PReLU, and Leaky-ReLU with accuracies of 98.3%, 100%, and 99.2%, respectively. In the work [39], a deep CNN-based capsule module (CNN-CapsNet) was implemented by utilizing a few residual blocks which classified 8-stages in magnetic resonance (MR) images of the TCGA-CESC database with an accuracy of 90.28%.

The study [40] deployed a TL-based exemplar pyramid model utilizing DarkNet19 or DarkNet53 for the retrieval of 21,000 attributes in which 1000 utmost-informatory were selected by neighborhood component analysis (NCA). SVM used these elected attributes for the classification of the LBC and SIPaKMeD databases and provided accuracies of 99.47% and 98.26%, respectively. In research [41], a novel deep architecture, HLDnet, was evolved that utilizes a faster RCNN network to detect HSIL+ cervigram images by dual-channel detection (acetic acid and Lugol's iodine cervigram). It achieved an accuracy of 0.86 for 400 training and validation and 200 tests, better than single-channel detection (either acetic acid or Lugol's iodine cervigram). There is more related work available in clinical practices [42–46] thanks to advances in computer vision methods. Some of these are observed as competent in performing the same or even better as the pathologists on medical data.

## 3. Material and Methods

### 3.1. Experimental Data

This work utilizes a publicly accessible SIPaKMeD database containing 966 WSI pap smear images and 4,049 images of handcrafted cropped cells [21]. An optical magnifying device (OLYMPUS BX53F) with a camera having a charge-coupled device (CCD) sensor (Lumenera's INFINITY-1) has been used to capture these pictures. The dataset is categorized into 5 classes by clinical professionals. The classes “superficial-intermediate (SI)” and “parabasal (P)” refer to “normal,” images sorted as “koilocytotic (K)” and “dyskeratotic (D)” indicate “abnormal,” and the remaining “metaplastic (M)” belongs to have “benign” cells. The experiment is performed on WSI slides and grouped into 5 class and 2 class (normal and abnormal).

Furthermore, the proposed framework is evaluated using liquid-based cytology (LBC) data available online at Mendeley data [22]. Based on the Bethesda system, the collection includes 963 WSI LBC high-resolution images organized into four sets of classes: “no squamous intraepithelial lesion (NILM),” “low-grade squamous intraepithelial lesion (LSIL),” “high-grade squamous intraepithelial lesion (HSIL),” and “squamous cell carcinoma (SCC).” The “NILM” indicates a “normal” grade, while the “LSIL,” “HSIL,” and “SCC” refer to “abnormal.”

### 3.2. Preprocessing

#### 3.2.1. Resizing and Division

The cervigram WSI images of both databases are of high resolution with 2048 × 1536 pixels. The images are resized to 224 × 224 pixels to reduce computation costs and make them fit into DL models. The resized data is get divided into a train, validation, and test data in the ratio of 3 : 1 : 1.

#### 3.2.2. Data Augmentation

DL models require a sufficient amount of data to train them efficaciously. Two-step data augmentation is utilized on the resized training data to increase the amount of data to be learned. Firstly, training data is augmented using a heavy augmentation pipeline consisting of numerous augmentation strategies such as affine transformations, perspective transformations, contrast changes, Gaussian noise, dropout of regions, hue/saturation changes, cropping/padding, and blurring. This augmentation pipeline, shown in Table 1, has 12 sets of augmentation functions that generated 12 augmented images for one training sample. A detailed description of these augmentations can be found in the ImgAug library [47].

Now, training data becomes 13 times more multiple than before. The structure of train, validation, and test data after resizing and augmenting SIPaKMeD and LBC data is given in Tables 2 and 3, respectively. Further, real-time data augmentation is performed using the “ImageDataGenerator” function of the Keras library that performs a random transformation on images so the model can be trained on distinct images on each epoch. The different arguments of the function are set to be: featurewise_center = false, rotation_range = 25, fill_mode = nearest, zoom_range = 0.2, width_shift_range = 0.1, height_shift_range = 0.1, horizontal_flip = true, vertical_flip = true, brightness_range = (0.5, 1.5), and channel_shift_range = 20.

### 3.3. Methods

#### 3.3.1. Feature Extraction Using Transfer Learning

**Figure 2:**
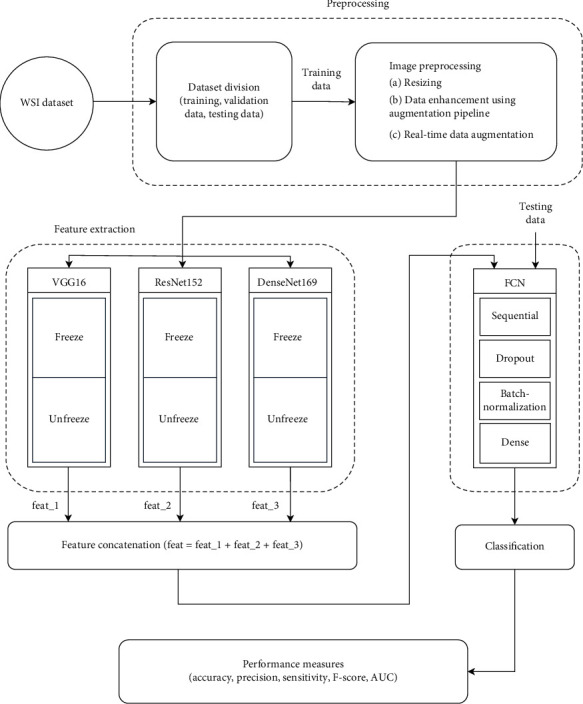
The overall architecture of the proposed method.

**Figure 3:**
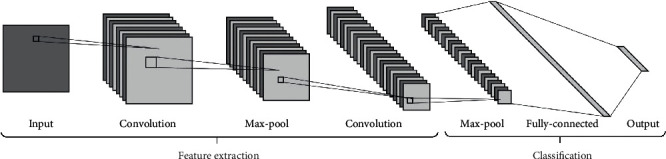
CNN model.

The overall architecture of the proposed method is shown in Figure 2. The preprocessed training data is being used for feature extraction using the fine-tuning of DL models. The DL models are certain subtypes of ML structures having more complex architecture based on a neural network with lesser human intercede [48]. These models are extensively utilized in extricating high-level features, providing progressive execution over the conventional approach, and expanding interpretability conjointly with the understanding and handling of biological information. Commonly used DL frameworks are CNNs, recurrent neural networks (RNNs), and recursive neural networks (RvNNs). Among these, CNN is the most prominent structure of DL having interconnected networks of neurons drafted in a combination of convolutional layers, pooling layers, and fully connected (FC) layers. Feature learning and classification are the prime utilization of the CNN model, as shown in Figure 3 [49].

The convolution layers extract a variety of visual features, including edges, objects, and textures by performing convolution of input and the kernel filter. The preactivation output *z^l^* of the convolution layer is defined as

(1) $$\begin{array}{c} z^{l}=v^{l-1}*W^{l} \end{array}$$

where *v*^*l*−1^ is the activation output of the previous layer, ∗ is the convolutional operator, and *W* is the weights. Subsequently, pooling layers include various operations like global average pooling, L-2 normalization, and max pooling to retain relevant features from the convolution layer output. Some of the pooling operators are given

(2) $$\begin{array}{c} \text{average}-\text{pool }{v_{xy}}^{l}=\frac{1}{{\text{s}}^{2}}\overset{s}{\underset{i,j}{\sum }}h_{\left(x+i\right)\left(y+j\right)}^{l-1}\\ \max-\text{pool }{v_{xy}}^{l}=\underset{i,j}{\max}h_{\left(x+i\right)\left(y+j\right)}^{l-1} \end{array}$$

The multidimensional feature map retrieved from these layers is used to convert it into a vector at the classification stage using the fully connected network (FCN). Here, in this work, three prevalent CNN-based DL methods VGG-16, ResNet-152, and DenseNet-169 are utilized for this purpose.

*VGG-16*. The Visual Geometry Group (VGG) was introduced with the concept of inserting a pile of small filters of size 3 × 3 instead of filters with sizes 5 × 5 and 11 × 11 in the preceding deep networks. Additionally, the convolution filter of size 1 ×1 with rectified linear unit (ReLU) activation function is inserted in between convolutional layers for complexity regulation and linear transformation. VGG-16 holds 16 layers, comprising convolution layers of kernel size 3 × 3 with padding of 1, and max pool layers of size 2×2 with a stride of 2, followed by three FC layers. This model has an input size of 224 ×224 × 3 with approx. 138 million computation parameters [50].

**Figure 4:**
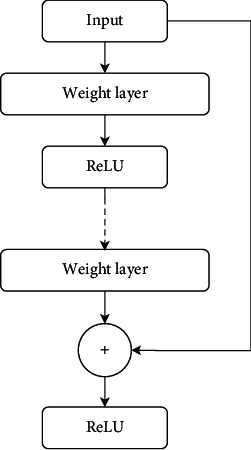
The residual block structure of ResNet.

*ResNet-152*. The residual network (ResNet) came with a more deep network by skipping connections to resolve the vanishing gradient issue. The substitute route connection made by the residual structure shown in Figure 4 permits the gradient to flow without fading, resulting in enhanced performance of the network. The residual behavior of ResNet for the *l*^th^ stage with *x* input and output *H* of the activation function can be formulated as

(3) $$\begin{array}{c} x_{l}=H_{l}\left(x_{l-1}\right)+x_{l-1}. \end{array}$$

The ResNet-152 model has 152 layers, more than 8 times deeper than VGG networks, nevertheless having a lesser computational complexity with approximately 60.4 million parameters [51].

**Figure 5:**
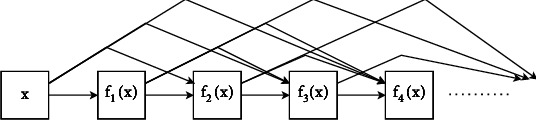
The densely connected structure of DenseNet.

*DenseNet-169*. This method is also used to resolve the issue of the vanishing gradient followed by the ResNet model. The contrast between ResNet and DenseNet is that ResNet utilizes an additive approach to associate all preceding feature maps, whereas DenseNet concatenates all the previous layers as shown in Figure 5. The value of variable x in DenseNet can be a map with the progressive complex group of functions as

(4) $$\begin{array}{c} \text{x}⟶\left[\text{x},{\text{f}}_{1}\left(\text{x}\right),{\text{f}}_{2}\left(\left[\text{x},{\text{f}}_{1}\left(\text{x}\right)\right]\right)+{\text{f}}_{3}\left(\left[\text{x},{\text{f}}_{1}\left(\text{x}\right),{\text{f}}_{2}\left(\left[\text{x},\text{f}1\left(\text{x}\right)\right]\right)\right]\right)\cdots ..\right]. \end{array}$$

**Figure 6:**
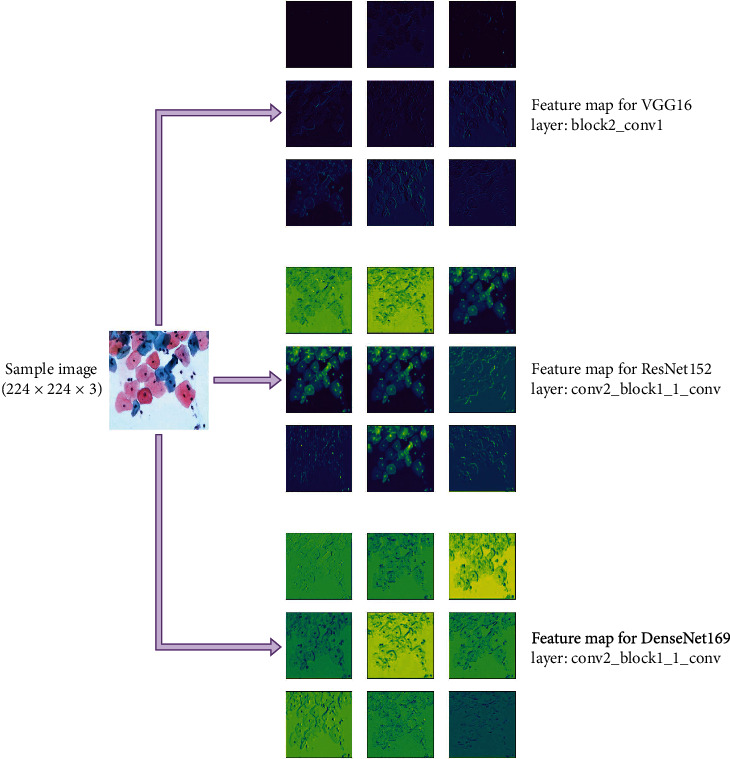
Visualization of feature maps for VGG-16, ResNet-152, and DenseNet-169.

The DenseNet-169 model comprises 169 layers, which is larger than the other members of the DenseNet group and has less computational complexity than the VGG and ResNet model with approximately 14.3 million parameters [52].  Figure 6 gives a visualization of feature maps for a sample image of SIPaKMeD data through a specific layer of the aforementioned DL models.

Performing image classification utilizing the DL approach requires plenty of computations that need the support of large data and long-running time of graphic processing unit (GPU) amid training of the model, which results in outlays of considerable computational resources and less productivity. TL accelerates the training process by utilizing the pre-trained model on a contemporary problem, which may result in more efficacy and overall accuracy. TL is substantially used as a design method in ML models to train with the small dataset by utilizing parameters of the pretrained model trained on one of some publicly available large datasets [53].

This paper utilizes the TL approach with the three DL models VGG-16, ResNet-152, and DenseNet-169 using pretrained weights on ImageNet datasets. The ImageNet dataset accommodates 1000 categories of objects with 1,281,167, 50,000, and 100,000 samples of training, validation, and testing instances, respectively. These models are fine-tuned by keeping some of the lower layers to be frozen and the higher layers kept unfrozen as shown in Figure 2. In this experiment, the lower layers of models VGG-16, ResNet-152, and DenseNet-169, up to layers “block4_pool,” “conv4_block29_out,” and “conv4_block1_0_relu,” respectively, are kept frozen, and the higher layers beyond this are being kept unfreeze. The weight parameters obtained by pretraining these models on the ImageNet dataset are utilized for fine-tuning the models by utilizing our training data of the pap-smear dataset. To extract the array of features some of the additional layers like global max pooling, batch normalization, dropout, and dense layers have been added to all three models which result in a bunch of 1024 features set from each model. All three models are trained on training data with a learning rate of 10^−4^ for 100 epochs, batch size of 32, and Adam optimizer. The batch size is set to 1 for validation and test data. The feature combinations “feat_1,” “feat_2,” and “feat_3” from each model are extracted in terms of weight files and saved as H5 extension files.

#### 3.3.2. Feature Concatenation

Each of the feature combinations extracted from the three fine-tuned models i.e. ‘feat_1', ‘feat_2', and ‘feat_3' has an array of 1024 features. These feature combinations are concatenated to produce hybrid features. Let there be two feature vectors v and w of dimensions n ×1 and m ×1, respectively, their concatenated feature vector having dimension k ×1. This concatenation of two feature vectors can be represented as:

(5) $$\begin{array}{c} z=v⨁w\;\text{where }v\in R^{n}\;\text{and }w\in R^{m} \end{array}$$

(6) $$\begin{array}{c} z={\left[v_{1},v_{2},\cdots ,v_{n}\right]}^{T}⨁{\left[w_{1},w_{2},\cdots ,w_{m}\right]}^{T} \end{array}$$

(7) $$\begin{array}{c} z={\left[v_{1},\cdots ,v_{n},w_{1},,\cdots ,w_{n}\right]}^{T}\in R^{n+m} \end{array}$$

(8) $$\begin{array}{c} f\left(z\right)\text{: }R^{n+m}⟶R^{k} \end{array}$$

Here ⨁ is called the concatenation operator. Each of the feature combinations ‘feat_1', ‘feat_2', and ‘feat_3' has a vector of size 1024 ×1, and the feature vector ‘feat' produced by concatenation of these feature combinations is having a size of 3072 ×1.

#### 3.3.3. Fully Connected Network (FCN)

Lastly, a segment of the FCN network has been implemented to perform the classification of cervical cancer test images. This network contains a sequential model with dropout, batch normalization, and dense (softmax activation function) layers. The input dimension of the FCN network is set to 3072 to feed the concatenated feature set “feat.” The output node of dense layers is set equal to the number of classes in the dataset. The output prediction “a_k_” for any test cervix image of class “*k*” is related as

(9) $$\begin{array}{c} a_{k}\epsilon \underset{i=1,.,k,.,n}{\sum }a_{i}. \end{array}$$

Here, *n* is the total number of classes in the cervical dataset. The experiment is performed by setting the learning rate to 10^−4^ for 300 epochs and a batch size of 1 with an Adam optimizer for testing of the data.

## 4. Results and Discussion

### 4.1. Implementation Environment

The proposed model is developed on a system with an operating system of Windows 10 having a graphic card from Nvidia Tesla V100 and 16 GB of GPU RAM. The experiment is performed on the Python 3.7 programming environment with requisite libraries like Scikit-learn, Tensorflow, Keras, Cuda, CuDNN, etc.

### 4.2. Performance Measures

The selection of the optimum classifier for best performance is achieved by choosing competent performance metrics. Accuracy, precision, recall, and F-score are some of the indices used in this research to assess the performance of the model [54]. Accuracy (ACC) in equation (10) gives the proportion of correctly predicted cases to the total number of cases. Precision (PRE) in equation (11) represents the fraction of correctly estimated positive patterns to all positively predicted patterns. Sensitivity (SEN) in equation (12) shows the rate of genuinely anticipated positive to all correctly classified instances. F-score (FS) in equation (13) formulates the harmonic average of precision (PRE) and sensitivity (SEN).

(10) $$\begin{array}{c} ACC=\frac{\text{TRPOS}+\text{TRNEG}}{\text{TRPOS}+\text{TRNEG}+\text{FALPOS}+\text{FALNEG}}, \end{array}$$

(11) $$\begin{array}{c} PRE=\frac{\text{TRPOS}}{\text{TRPOS}+\text{FALPOS}}, \end{array}$$

(12) $$\begin{array}{c} SEN=\frac{\text{TRPOS}}{\text{TRPOS}+\text{FALNEG}}, \end{array}$$

(13) $$\begin{array}{c} FS=\frac{2*\text{PRE}*\text{SEN}}{\text{PRE}+\text{SEN}}, \end{array}$$

where TRPOS is the true positive, TRNEG is the true negative, FALPOS is the false positive, and FALNEG is the false negative.

### 4.3. Results

**Figure 7:**
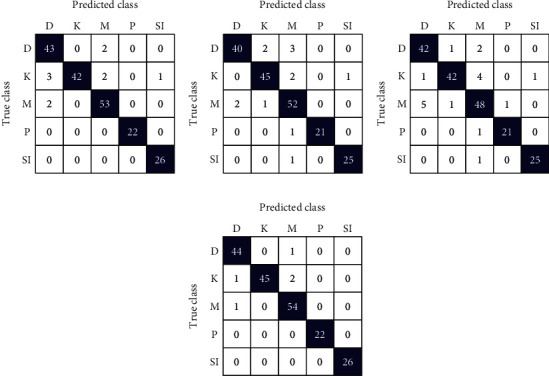
Confusion matrix for SIPaKMeD WSI 5-class classification using (a) VGG-16, (b) ResNet-152, (c) DenseNet-169, and (d) proposed model.

**Figure 8:**
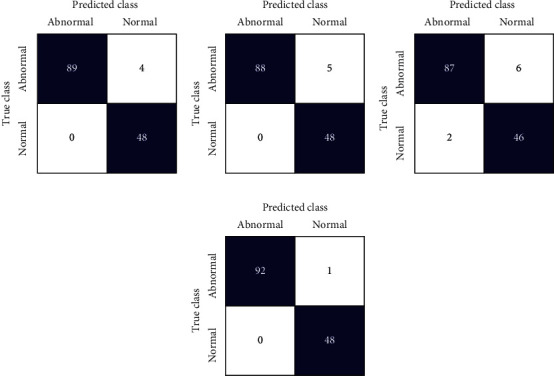
Confusion matrix for SIPaKMeD WSI 2-class classification using (a) VGG-16, (b) ResNet-152, (c) DenseNet-169, and (d) proposed model.

This section shows the outcomes of the experiment performed on WSI SIPaKMeD cervical cancer data. Here, the implementation of the proposed model has also been analyzed on another cervical cancer database, i.e., LBC data. The performance is evaluated for individual fine-tuned models such as VGG-16, ResNet-152, and DenseNet-169 classifiers as well as for the proposed model. Figures 7 and 8 show the confusion matrix obtained for the 5-class and 2-class classifications of WSI SIPaKMeD data for the different classifiers. Both the figures describe that the confusion matrix obtained for the proposed model predicts the least incorrect instances.

**Figure 9:**
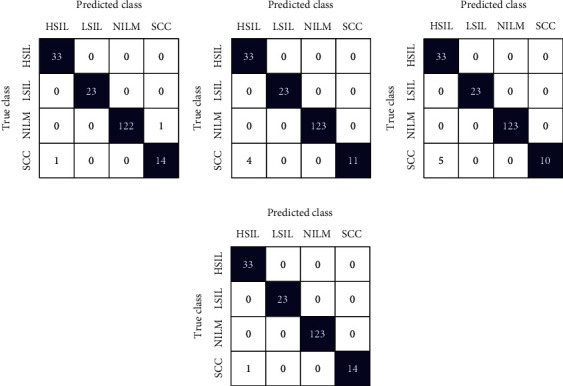
Confusion matrix for LBC WSI data classification using (a) VGG-16, (b) ResNet-152, (c) DenseNet-169, and (d) proposed model.

**Figure 10:**
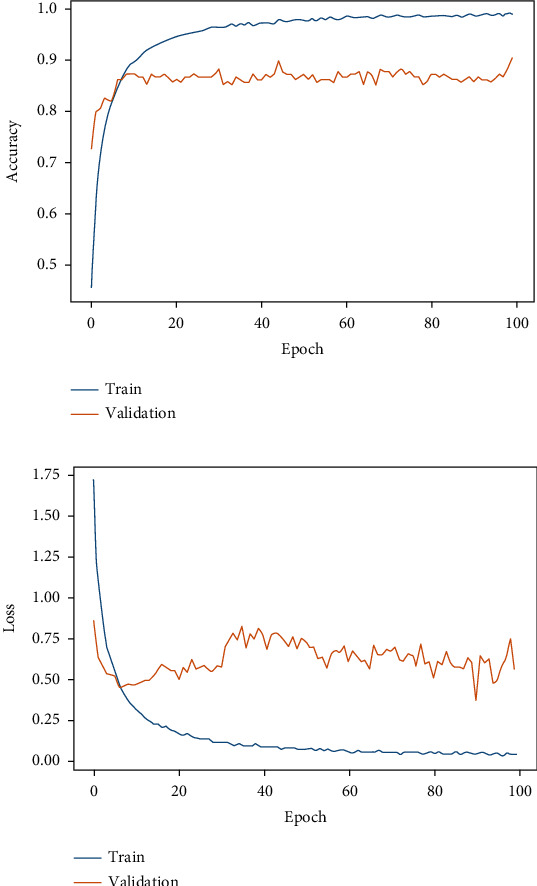
Fine-tuned VGG-16. (a) Accuracy curve. (b) Loss curve for SIPaKMeD 5-class classification.

**Figure 11:**
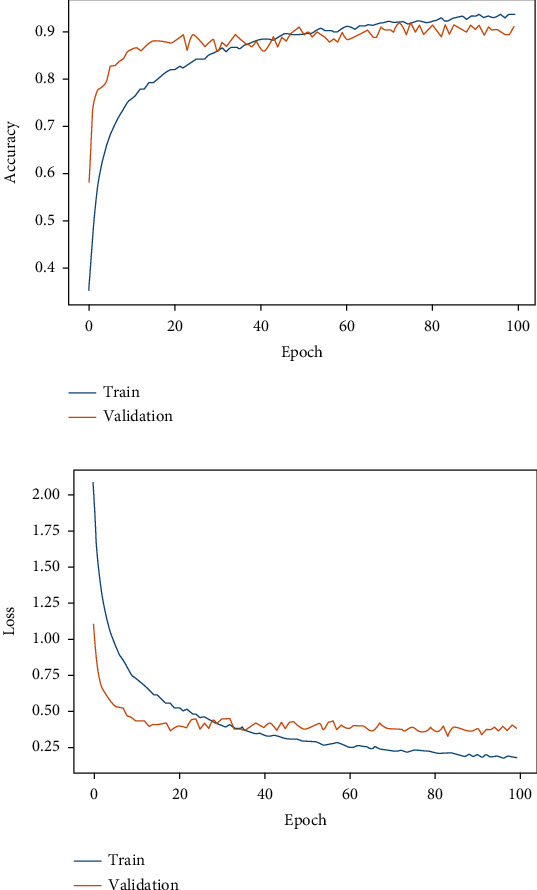
Fine-tuned ResNet-152. (a) Accuracy curve. (b) Loss curve for SIPaKMeD 5-class classification.

**Figure 12:**
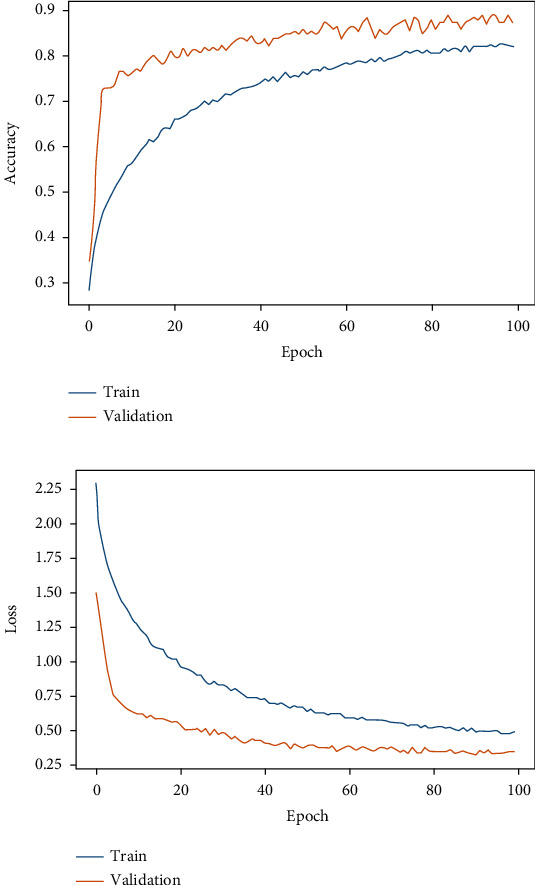
Fine-tuned DenseNet-169. (a) Accuracy curve. (b) Loss curve for SIPaKMeD 5-class classification.

**Figure 13:**
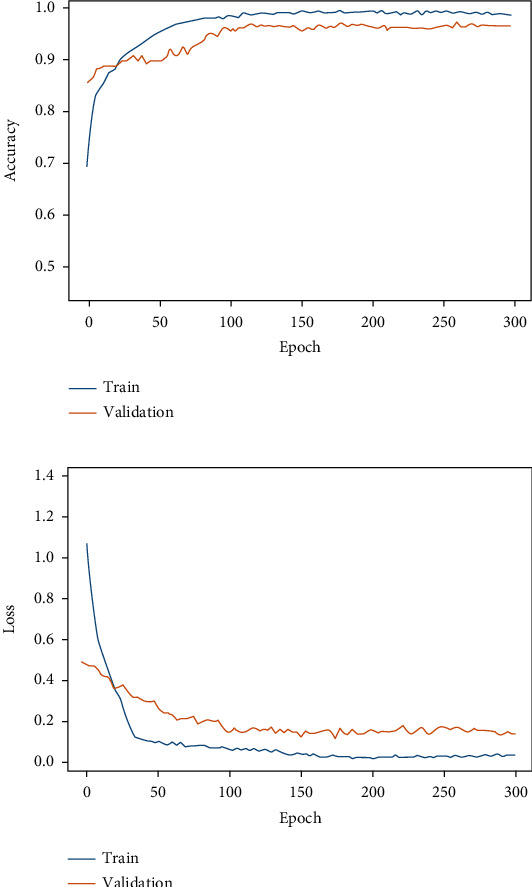
Proposed model. (a) Accuracy curve. (b) Loss curve for SIPaKMeD 5-class classification.

Table 4 depicts the results obtained from different classifiers and our proposed model on SIPaKMeD WSI data for classification into 5 class and 2 class. Here is the performance of the individual fine-tuned models of VGG-16, ResNet-152, and DenseNet-169 are confronted with our proposed HDFCN model. Our proposed model achieves better results than the other classifiers with an accuracy of 97.45% and 99.29% for 5-class and 2-class classifications, respectively. For 5-class classification, our model predicts the pap-smear images with a precision score of 97.94%, a recall value of 98.08%, and an F-score of 98.01%. This model outperforms the other classifiers with the precision, recall, and F-score values of 98.92%, 100%, and 99.46%, respectively, in 2 class classification. Furthermore, the proposed model is assessed for LBC WSI pap-smear data. The confusion matrix and the performance indices obtained for the baseline fine-tuned classifiers and the proposed model are shown in Figure 9 and Table 5, respectively. The accuracy-loss curve for 5-class classification of SIPaKMeD pap smear data is shown in Figures 10–13. Observation shows that the proposed model achieves excellent results in classifying this data with the accuracy, precision, recall, and F-score value of 99.49%, 98.33%, 99.26%, and 98.79%, respectively.

### 4.4. Discussion

#### 4.4.1. Comparative Analysis

Table 6 represents the comparison of the proposed model with the existing state-of-the-art methods for 5-class and 2-class classifications of SIPaKMeD WSI data. The comparison review of the performance of the proposed model for LBC WSI data is given in Table 7. These comparisons convincingly conclude the preciseness of the framework in cervical cancer classification.

#### 4.4.2. McNemar's Test

Here, we performed McNemar's statistical non-parametric test on the baseline classifiers and our proposed model [55].  Table 8 shows the *p* value calculated through McNemar's test between the individual baseline classifiers (VGG-16, ResNet-152, and DenseNet-169) and the proposed model. This test signifies the fact that the individual baseline classifiers and the proposed model are dissimilar in extracting features if the *p* value computed is less than 5% or 0.05. As this *p* value is calculated below 0.05 for all the cases on both the datasets utilized, the null hypothesis is false, and the proposed classifier is dissimilar to the other DL classifier with enhanced performance. This scrutiny proves the reliability and authenticity of the framework we proposed in the classification of cervical cancer WSI pap-smear data.

## 5. Conclusion and Future Work

The escalating use of computer vision models in the early-stage detection of cervical cancer motivates us to propose this hybrid framework. The proposed work utilizes two-step data augmentation to increase the amount of training data. The proposed HDFCN model utilizes the hybrid features obtained from the concatenation of features extracted from the fine-tuned models of three prevalent DL algorithms: VGG-16, ResNet-152, and DenseNet-169. These hybrid features are used for the classification of cervical cancer WSI pap-smear data. The proposed model is evaluated on SIPaKMeD data and gives an accuracy of 97.45% for 5-class classification and 99.29% for 2-class classification. Moreover, the experiment performed on LBC WSI data provides 99.49% accuracy. The precise recognition of infected WSI images enables experts to perform a more in-depth analysis of cells within the images. The futuristic approach to this method involves the utilization of more optimal feature selection algorithms, progressive resizing, and advanced ensemble methods to further improve model performance and computation cost-cutting.

## Figures and Tables

**Table 1:** Pipeline of augmentation functions.

S.no.	Aug_function	Augmentations
1	Aug	Rotate (-45, 45), scale {“*x*”: (0.8, 1.2), “*y*”: (0.8, 1.2)}, translation {“*x*”: (-0.15, 0.15), “*y*”: (-0.15, 0.15)}, shear (-2, 2), h_flip (1.0), v_flip (1.0)
2	Clahe	clip_limit (1, 10), tile_grid_size (3, 21), gamma_contrast (0.5, 2.0), channel_clahe
3	Edge	edge_detect (alpha = (0, 0.5), directed_edge_detect (alpha = (0, 0.5), direction = (0.0, 1.0))
4	Sharp	Sharpen (alpha = (0.2, 0.8), lightness = (0.75, 1.5))
5	Canny	Canny (alpha = (0.5, 0.8), sobel_kernel_size = (3, 7))
6	Red	red_channel (add ((10, 100), rotate = (0, 45))
7	Green	green_channel (add ((10, 100), rotate = (0, 45))
8	Blue	blue_ channel (add ((10, 100), rotate = (0, 45))
9	Noise	Blur (sigma = (0, 1.22), gauss_noise (scale = 0.111^∗^255), laplace_noise (scale = (0, 0.111^∗^255))
10	Color	channel_shuffle (1.0), grayscale (1.0), hue_n_saturation (0.5, 1.5), add_hue_saturation (-50, 50), kmeans_color (n_colors = (4, 16)
11	Flip	histogram_equalization, v_flip (1.0), h_flip (1.0)
12	contrast_n_shit	Contrast (LinearContrast (0.75, 1.5)), brightness (0.35, 1.65), brightness_channel ((0.5, 1.5), per_channel = 0.75)

**Table 2:** Structure of augmented SIPaKMeD data.

Class	Train	Validation	Test
SI	975	25	26
P	832	22	22
K	1846	48	48
M	2106	54	55
D	1729	45	45
Total	7488	194	196

**Table 3:** Structure of augmented LBC data.

Class	Train	Validation	Test
NILM	4758	123	123
LSIL	871	23	23
HSIL	1261	33	33
SCC	572	15	15
Total	7462	194	194

**Table 4:** Performance metrics for different fine-tuned classifiers and proposed model on SIPaKMeD WSI data.

Data	Models	Accuracy (%)	Precision (%)	Recall (%)	F-score (%)
SIPaKMeD WSI 5-class	VGG-16	94.89	95.88	95.77	95.83
	ResNet-152	93.37	93.76	94.66	94.21
	DenseNet-169	90.82	91.94	92.06	92
	Proposed model	**97.45**	**97.94**	**98.08**	**98.01**

SIPaKMeD WSI 2-class	VGG-16	97.16	95.7	100	97.8
	ResNet-152	96.45	94.62	100	97.24
	DenseNet-169	94.33	93.55	97.75	95.6
	Proposed model	**99.29**	**98.92**	**100**	**99.46**

Best results are shown in bold.

**Table 5:** Performance metrics for different fine-tuned classifiers and proposed model on LBC data.

Data	Models	Accuracy (%)	Precision (%)	Recall (%)	F-score (%)
LBC WSI data	VGG-16	98.97	98.13	97.59	97.86
	ResNet-152	97.94	93.33	97.29	95.27
	DenseNet-169	97.42	91.67	96.71	94.12
	Proposed model	**99.49**	**98.33**	**99.26**	**98.79**

Best results are shown in bold.

**Table 6:** Comparison of classification accuracy for the proposed model with previous methods on SIPaKMeD WSI data.

Methods	Classification accuracy (%)	
	5 class	2 class
Ensemble [56]	94.09	98.27
CNN+PCA [57]	96.37	—
Fuzzy-based ensemble [58]	95.43	98.55
RCAN-DenseNet-121 [59]	91.09	—
ResNet-50 [60]	91	—
Wavelet+CNN+RF [61]	97.01%	—
Proposed model	**97.45**	**99.29**

Best results are shown in bold.

**Table 7:** Comparison of classification accuracy for the proposed model with previous methods on LBC WSI data.

Methods	Classification accuracy (%)
Fuzzy-based ensemble [58]	99.23
CNN [62]	96.89
T2T-ViT+Transfer learning [63]	98.79
Exemplar Pyramid+NCA+SVM [40]	99.47
Proposed model	**99.49**

Best results are shown in bold.

**Table 8:** McNemar's test result (*p* value).

McNemar's test for the proposed model and the baseline classifier as	*p* value	
	SIPaKMeD WSI data	LBC WSI data
VGG-16	0.0007	0.0196
ResNet-152	0.0008	0.0012
DenseNet-169	0.0106	0.0073

*p* < 0.5 conclude false null hypothesis.

## Data Availability

The data used to support the findings of this study are included in the article.
